# Dynamics of the Human Nasal Microbiota and Staphylococcus aureus CC398 Carriage in Pig Truck Drivers across One Workweek

**DOI:** 10.1128/AEM.01225-21

**Published:** 2021-08-26

**Authors:** Anna Cäcilia Ingham, Tinna Ravnholt Urth, Raphael Niklaus Sieber, Marc Stegger, Sofie Marie Edslev, Øystein Angen, Anders Rhod Larsen

**Affiliations:** a Department of Bacteria, Parasites and Fungi, Statens Serum Institut, Copenhagen, Denmark; b Department of Infectious Disease Epidemiology and Prevention, Statens Serum Institut, Copenhagen, Denmark; University of Manchester

**Keywords:** LA-MRSA CC398, MRSA transmission, *Staphylococcus aureus*, nasal microbiota, microbiome, pig transport, swine lorry

## Abstract

Drivers of pig trucks constitute a potential route of human transmission of livestock-associated methicillin-resistant Staphylococcus aureus clonal complex 398 (LA-MRSA CC398). In this study, we determined MRSA prevalence in pig truck drivers (*n* = 47) and monitored the nasal microbiota of 9 drivers 3 times daily throughout 1 workweek (*n* = 113 samples) and compared it to that of their spouses (*n* = 25 samples from 6 spouses) and 89 nonexposed subjects. S. aureus isolates (*n* = 232) derived from a subset of nasal and truck samples were whole-genome sequenced. The nasal alpha diversity of drivers in the beginning of the workday was lower than that of nonexposed subjects. During the workday, it increased significantly. Similarly, the drivers’ nasal composition shifted during the workday, becoming increasingly different from that of their spouses and nonexposed individuals. Clustering into community state types (CSTs) revealed frequent switches from either S. aureus- or *Corynebacterium*-dominated CSTs in the mornings to a *Psychrobacter-*dominated CST during the workday. Six intermittent MRSA carriers were mostly MRSA negative in the mornings, and their nasal microbiota resembled that of nonexposed subjects. When acquiring MRSA during the workday, they switched to the *Psychrobacter-*dominated CST. In contrast, the nasal microbiota of two persistent MRSA carriers was dominated by staphylococci. In conclusion, we show that the nasal microbiota of pig truck drivers is very dynamic, undergoes drastic changes during workdays, and differs from that of nonexposed subjects even before pig contact. MRSA-carrying drivers may eventually introduce MRSA into the community and health care facilities. Carriage dynamics, however, showed that for most drivers, CC398 MRSA is rapidly lost and only rarely causes transmission to spouses.

**IMPORTANCE** In Denmark, the number of human methicillin-resistant Staphylococcus aureus (MRSA) cases has increased dramatically since the early 2000s, starting from imported cases and spreading in the community. However, today, approximately one-third of all new cases are attributed to livestock-associated MRSA clonal complex 398 (LA-MRSA CC398). This mirrors the increase in pig farms, of which 95% are now positive for LA-MRSA, and this has been caused mainly by three dominant lineages enriched for a number of key antimicrobial resistance genes. Although most human LA-MRSA CC398 infections in Denmark are linked to livestock contact, still up to one-third are not. Pig truck drivers constitute a previously understudied occupation group which may transmit LA-MRSA CC398 to household members, the community, and hospitals. In this study, we demonstrate dramatic work-related changes in the nasal microbiota of pig truck drivers, as well as in their carriage of LA-MRSA CC398. However, they likely do not constitute an important reservoir for LA-MRSA CC398 dissemination.

## INTRODUCTION

In Europe, the pig industry constitutes the main reservoir for livestock-associated methicillin-resistant Staphylococcus aureus clonal complex 398 (LA-MRSA CC398) (1). Transmission of LA-MRSA from animals to humans has been of great concern in some European countries, especially in those with low MRSA incidence and large pig productions. The level of LA-MRSA-positive Danish swine herds had in 2019 reached 95%, which was mainly caused by three dominant lineages (L1 to L3) enriched for a number of antimicrobial resistance genes, such as *tet*(K) and *tet*(M) (both tetracycline resistance), *lnu*(B) (lincosamide resistance), *czrC* (cadmium/zinc resistance), and *str* (aminoglycoside resistance) (2, 3). At the same time, the number of human LA-MRSA CC398 cases increased over the last decade (2). Of all 3,657 human MRSA cases in Denmark in 2019, 1,122 cases (31%) were LA-MRSA CC398, and of those cases, 993 (89%) occurred in patients with contact to livestock production (2). However, two studies monitoring LA-MRSA CC398 infections in Denmark from 1999 to 2011 and 2010 to 2015, respectively, found that approximately one-third of cases were not associated with livestock contact (4, 5).

Direct contact with swine is regarded as a personal risk factor that is asked about at hospital admissions in Denmark. The spread to the general public is a major concern, as LA-MRSA can be introduced to hospitals by infected or colonized persons with no livestock contact. Transmission studies have shown that humans are transiently contaminated by LA-MRSA after a short-term visit to a swine farm and that this is correlated with the amount of MRSA in the air but also that the level of contamination declines rapidly to unquantifiable levels after a few hours (6). Pig farm workers, however, may carry LA-MRSA CC398 for several weeks outside the farm environment (7).

The movement of pigs between farms has been shown to be a major factor in the rapid spread of LA-MRSA between swine farms (3). Transmission between farms has also been shown to take place due to insufficiently cleaned trucks (8, 9). A group that has not received much attention in the pig production system until now is the pig truck drivers. Among all LA-MRSA CC398 cases registered in the Danish National MRSA database at Statens Serum Institut, Copenhagen, pig truck drivers constituted 1% (*n* = 71) between January 2006 and June 2019. They visit different swine farms and have close physical contact with the animals both at the swine farm and at the slaughterhouse for several hours each day. Therefore, it could be expected that the nasal microbiota of pig truck drivers is highly influenced by the microbiota found in swine farms, similar to what has been shown earlier for farm workers (10, 11). Pig truck drivers might therefore also end up as persistent carriers of LA-MRSA. Persistent nasal carriers often harbor S. aureus at higher loads and for longer periods and have an increased risk of S. aureus infection compared with intermittent carriers or noncarriers (12). Pig truck drivers consequently could have a role in the transmission of MRSA between pig farms but also in the transmission of MRSA into the general population. S. aureus carriage has been shown to be highly dependent on the composition of the prevailing nasal bacterial community, which itself is impacted by pig exposure and could be enriched for further livestock-associated pathogens (10–12).

In this study, the prevalence of LA-MRSA carriage among pig truck drivers was determined. For a subgroup of drivers and their spouses, we monitored MRSA lineage dynamics and changes in the nasal microbiota three times daily for one working week. At the same time, MRSA was also isolated from the pig trucks and subjected to whole-genome sequencing (WGS) in order to compare the strains with those isolated from the nasal cavity of the truck drivers. The aim of the study was to describe the changes taking place in the human nasal microbiota of pig truck drivers in relation to MRSA colonization dynamics.

## RESULTS

### Prevalence of LA-MRSA CC398 carriage in pig truck drivers.

From the initial screening of 47 pig truck drivers, 19 (40%) were MRSA positive with a median value of 180 CFUs of MRSA/swab. Five of the drivers (11%) had CFU values above 1,000 MRSA/swab. All MRSA isolates belonged to CC398 with *spa* types t011 and t034.

Nine drivers who had tested positive at the initial screening volunteered to participate in a follow-up study for 1 workweek. Among these nine truck drivers, one was MRSA negative at all subsequent samplings (driver 01), two were MRSA positive at all subsequent samplings (22%; driver 04 and driver 07), and the remaining six (67%) were intermittently MRSA positive. Results from MRSA cultivation from the nine drivers and their spouses and trucks are shown in Table 1. All MRSA isolates from the subset of nine drivers belonged to CC398.

**TABLE 1:** MRSA carriage in drivers, spouses, and trucks in nine households^*a*^

Household	Data for:										
	Drivers			Spouses			Trucks				
	No. of available samples	No. of MRSA-positive samples (%)	Avg CFU/ml in positive samples	No. of available samples	No. of MRSA-positive samples (%)	Avg CFU/ml in positive samples	No. of available samples	No. of MRSA-positive samples (%)		Avg CFU/ml in positive samples	
1	14	0 (0)		0			9	5 (56)		212
2	13	8 (62)	264	4	0 (0)		9	9 (100)		350
3	9	8 (89)	562	0			9	4 (44)		128
4	10	10 (100)	2376	0			5	4 (80)		170
5	13	6 (46)	573	2	0 (0)		7	2 (29)		218
6	15	7 (47)	63	4	0 (0)		10	3 (30)		13
7	12	12 (100)	>10^5^	5	3 (60)	>10^5^	7	3 (43)		67
8	13	5 (38)	761	5	0 (0)		8	1 (13)		310
9	14	13 (93)	>10^5^	5	1 (20)	800	9	4 (44)		155
Total	113			25			73					

a Drivers from households 4 and 7 were persistent MRSA carriers. Samples from drivers were collected 3 times daily across 1 workweek (6 days) at 15 time points in total. Truck samples were collected 2 times daily across 5 days, at 10 time points in total. Samples from spouses were collected each morning across 5 days, i.e., at 5 time points in total.

### Nasal bacterial alpha diversity increases during the truck drivers’ workday.

**FIG 1:**
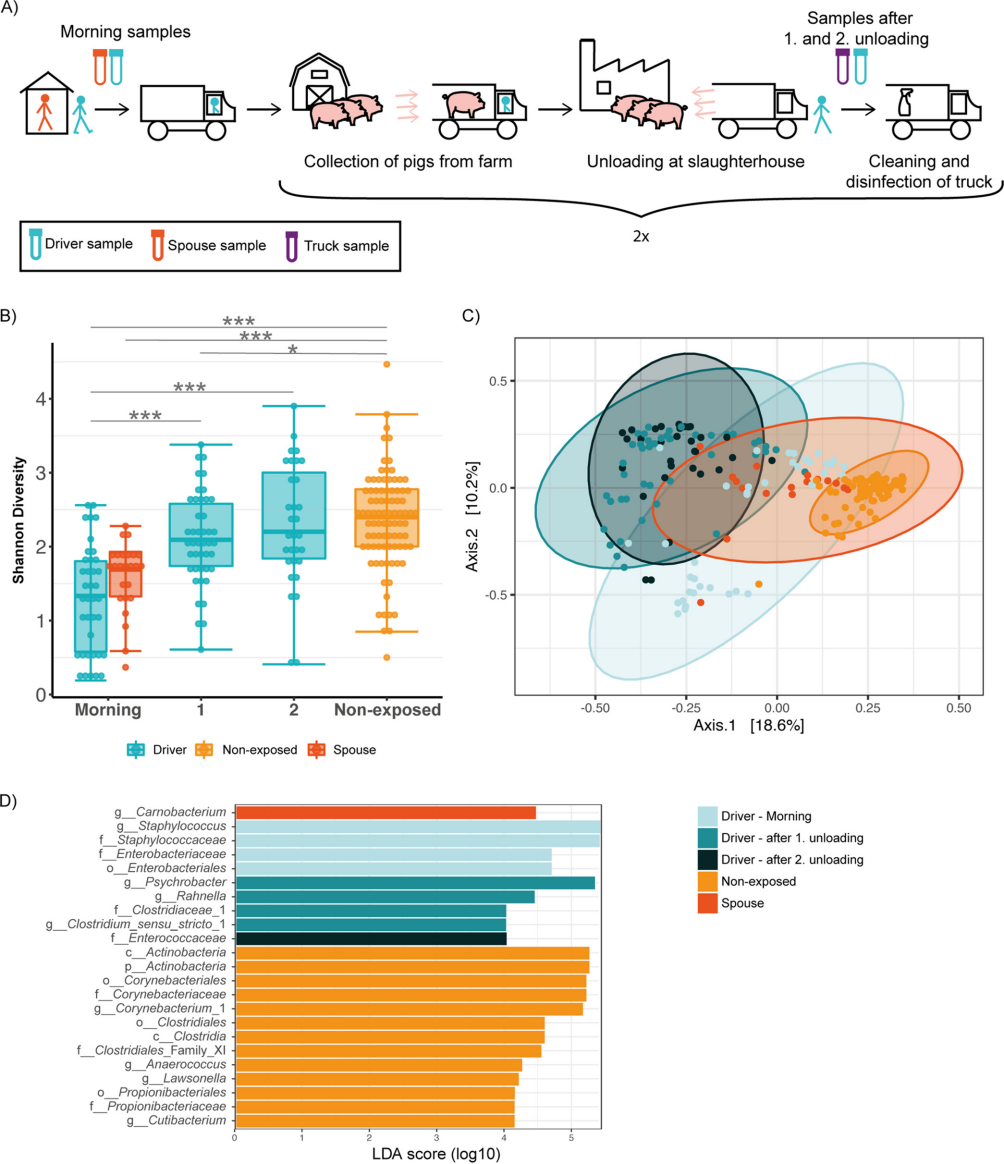
Study outline and alpha and beta diversity of the nasal microbiota. (A) Study outline showing sampling time points during one workday of pig truck drivers. Swabs were collected from Monday to Friday of one workweek, as well as the following Monday morning. (B) Nasal bacterial alpha diversity across the workday by means of the Shannon index. Each time of day contains repeated measures from the nine drivers across the six workdays of the monitored week. (C) Principal-coordinates analysis of the nasal bacterial community (beta diversity) showing differences between time points during the workday and differences between drivers, spouses, and nonexposed individuals. (D) Linear discriminant analysis (LDA) effect size (LEfSe) identified differentially abundant taxa between time points during the drivers’ workday and between drivers, spouses, and nonexposed individuals. The higher the LDA score (log_10_), the higher the effect size of the respective taxon in explaining the group difference. Here, we show taxa with an LDA score of >4. Abbreviations: 1, time point after first unloading; 2, time point after second unloading; p, phylum; c, class; o, order; f, family; g, genus. Asterisks indicate the following significance levels: *, *P* < 0.05; **, *P* < 0.01; and ***, *P* < 0.001.

Across one workweek, we used 16S rRNA gene sequencing to characterize the nasal microbiota of nine pig truck drivers three times daily (i) in the morning before work, (ii) after the first unloading of pigs, and (iii) after the second unloading of pigs (Fig. 1a). In addition, the spouses of six drivers were sampled each morning.

In order to compare bacterial richness and evenness between drivers, spouses, and nonexposed adults, as well as investigate temporal variation, we estimated bacterial alpha diversity using the Shannon index. Drivers and their spouses exhibited similar levels of nasal bacterial alpha diversity in morning samples (Fig. 1b). Drivers' alpha diversities at the three daily time points did not differ by weekday (see Fig. S1 in the supplemental material). However, across the workday, we observed a recurring strong pattern (Fig. 1b and Fig. S1), as follows: alpha diversity increased significantly from before work in the morning to after the first and second unloading of pigs (*P* < 0.001) (Fig. 1b). Nonexposed individuals had significantly higher nasal bacterial diversity than morning samples of both drivers and their spouses (*P* < 0.001), as well as compared with drivers after the first unloading (*P* = 0.022) (Fig. 1b).

### Nasal bacterial composition changes during the truck drivers’ workday.

Next, we aimed at investigating differences in the bacterial community structure between samples, i.e., beta diversity. Therefore, we visualized dissimilarities in the nasal bacterial composition of drivers at the three daily time points, as well as of their spouses and of the nonexposed subjects in a principal-coordinate analysis (PCoA) (Fig. 1c). The drivers’ bacterial community structure in the morning differed significantly from that of their spouses (analysis of similarity [ANOSIM], *P* = 0.001, *R* = 0.186) (Fig. 1c).

The bacterial community in the drivers’ noses in the morning was significantly different from that found after the first unloading (ANOSIM, *P* = 0.001, *R* = 0.36), as well as after the second unloading of pigs (ANOSIM, *P* = 0.001, *R* = 0.37) (Fig. 1c). However, morning samples had a higher within-group variation, i.e., they differed more from each other than samples after first unloading. The PCoA indicated that there was an overlap in composition between morning samples and samples after first unloading, but a subgroup of morning samples differed from samples of the following time point (Fig. 1c). The composition changed significantly from the first to the second unloading as well (ANOSIM, *P* = 0.017, *R* = 0.054) (Fig. 1c). Although, the difference of mean ranks (ANOSIM statistic R, see Materials and Methods) was almost 1 order of magnitude smaller than in the comparisons with the morning samples. Moreover, samples after second unloading had a high within-group variation.

Nonexposed subjects differed significantly from drivers’ spouses (*P* = 0.001, *R* = 0.57), as well as from drivers in the morning (*P* = 0.001, *R* = 0.66), after first unloading (*P* = 0.001, *R* = 0.9) and after second unloading (*P* = 0.001, *R* = 0.94).

Even though nonexposed subjects differed in nasal bacterial composition from both spouses and drivers at all daily time points, the difference of mean ranks (ANOSIM statistic R) was smallest between nonexposed subjects and spouses. Comparing the bacterial composition of nonexposed subjects to drivers, the difference of mean ranks (ANOSIM statistic R) increased across the workday (Fig. 1c).

After showing that alpha and beta diversity changed during the drivers’ workday and differed from nonexposed individuals, we aimed to identify distinct taxonomical profiles reflecting these changes. Through linear discriminant analysis effect size (LEfSe), we found taxa of several taxonomic levels in the drivers’ anterior nares that were significantly differentially abundant between time points during the workday (Fig. 1d). Staphylococcus was overrepresented in the morning (*P* < 0.001) (Fig. 1d). Genera overrepresented after the first unloading of pigs included *Psychrobacter*, *Rahnella*, and *Clostridium sensu stricto* (all *P* < 0.001), while *Enterococcaceae* (*P* < 0.001) characterized samples after the second unloading (Fig. 1d). Comparing the nasal taxonomic profiles of drivers to those of their spouses and nonexposed individuals, we observed that spouses were characterized by a high abundance of *Carnobacterium* (*P* < 0.001) (Fig. 1d). The genera *Corynebacterium*, *Anaerococcus*, *Lawsonella*, and *Cutibacterium* were significantly overrepresented in nonexposed individuals (all *P* < 0.001) (Fig. 1d). A complete list of the differentially abundant taxa identified by LEfSe is provided in Table S1 in the supplemental material.

### Absolute bacterial load increases during the workday.

We employed 16S rRNA (V3-V4 region) quantitative PCR (qPCR) to assess absolute bacterial abundance in the nasal samples, defined as 16S rRNA gene copies per sample (see Fig. S2a in the supplemental material). The overall bacterial load in driver samples increased significantly from a median of 9.3 gene copies per sample (log_10_) in the morning to 9.5 after first (*P* = 0.05) and 9.8 after second unloading (*P* = 0.01), respectively (Fig. S2a). Bacterial load of driver samples in the morning also differed significantly from that of spouse samples (median of 8.8 gene copies per sample [log_10_], *P* = 0.02). Nonexposed individuals had a considerably lower absolute bacterial abundance of a median 6.7 gene copies per sample (log_10_) than drivers and spouses.

The absolute abundance of the genus Staphylococcus per sample was estimated from sample-wise proportions (Fig. S2b). We observed a slight decrease, although not significant, in drivers’ nasal staphylococcal load during the workday from a median 9.1 gene copies per sample (log_10_) in the morning to 8.6 and 8.5 after first and second unloading (Fig. S2b), respectively. Spouses had a significantly lower staphylococcal load than drivers in the morning (median of 8.2 gene copies per sample [log_10_], *P* = 0.01).

### Community state typing supports distinct taxonomic patterns and temporal dynamics.

**FIG 2:**
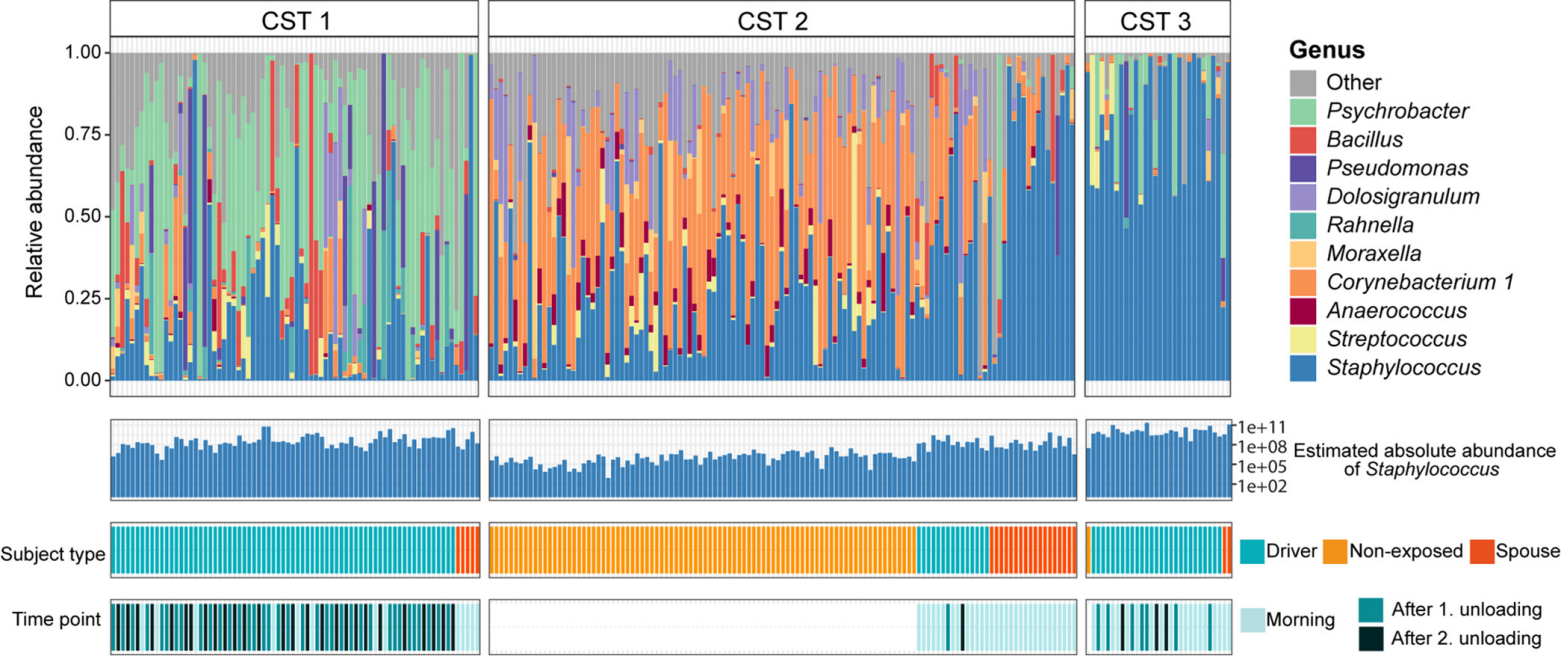
Community state types of the nasal community. Relative abundance of the 10 most abundant genera across the data set per CST. The panel in the second row shows sample-wise estimated absolute abundance of Staphylococcus, assessed via 16S rRNA qPCR. The third and fourth panel indicate group affiliation (driver, nonexposed, or spouse) and time point of sample collection (morning and after first or second unloading), respectively. Abbreviations: CST, community state type.

In addition to differential abundance analysis, we assessed taxonomic clustering in an approach naive for *a priori* grouping information. We grouped all samples into community state types (CSTs) by partitioning around medoid (PAM) clustering and identified three CSTs (Fig. 2). The most abundant genera (based on average relative abundance) in CST 1 were *Psychrobacter* (35%), Staphylococcus (17%), *Bacillus* (7%), and Pseudomonas (7%). CST 2 was characterized by high relative abundances of Staphylococcus (33%), *Corynebacterium* (30%), and *Dolosigranulum* (8%). Staphylococcus clearly dominated CST 3 with 80% average relative abundance, followed by *Psychrobacter* (9%). In agreement with this finding, absolute staphylococcal abundance was highest in CST 3 samples, followed by CST 1 and CST 2 (median gene copies per sample [log_10_] of 9.9, 8.4, and 6.4, respectively) (Fig. 2). Staphylococcal species assignment of the 16S rRNA gene sequences was ambiguous. However, a BLAST search of the overall most abundant staphylococcal amplicon sequence variants (ASVs) revealed that staphylococcal dominance in CST 3 was probably attributable to S. aureus (ASV1; 80% median relative abundance in CST 3 samples) (see Fig. S3 in the supplemental material). In contrast, Staphylococcus epidermidis was most likely the most abundant staphylococcal species in CST 2 (ASV2; 13% median relative abundance in CST 2 samples) (Fig. S3).

Community state typing confirmed that the bacterial composition differed between time points within a workday. Drivers’ morning samples were represented in all three CSTs (21% CST 1, 34% CST 2, and 45% CST 3). Samples after the first and second unloading were primarily affiliated with CST 1 (82% and 87%, respectively). This result is in congruence with our comparison of beta diversity between morning samples and samples after first unloading which suggested that a subset of morning samples, i.e., those affiliated with CST 1, resembled samples after first and second unloading in composition. Most morning samples from spouses (72%), as well as the vast majority of samples from nonexposed individuals (99%), belonged to CST 2 (Fig. 2).

**FIG 3:**
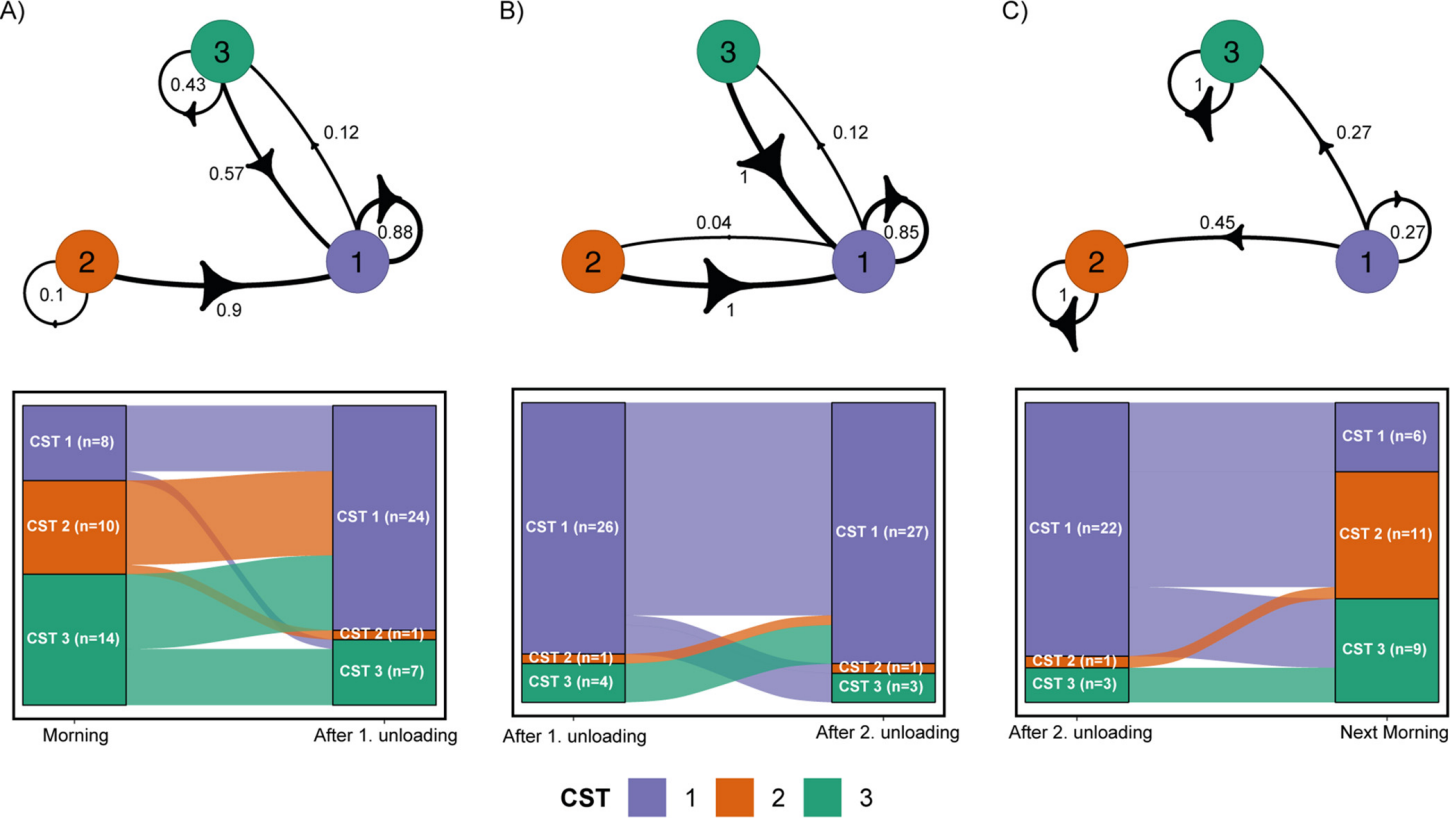
CST transitions across the drivers’ workday. (A) Transitions from morning to first unloading displayed by Markov chains and Sankey diagrams of drivers’ nasal bacterial CST transitions across the workday (collapsed by weekday). Arrows in Markov chains indicate whether a driver switches from one CST to another or remains within the same CST at the following time point. Arrow annotations display the proportion of drivers undergoing each transition, which is also represented by the arrow width. Sankey diagrams contain only complete pairs of two consecutive samples to be comparable to the Markov chains. (B) Transitions from first to second unloading. (C) Transitions from second unloading to next morning, with the exception of the last transition from Friday after second unloading to the following Monday morning. Abbreviations: CST, community state type.

We used Markov chains to show transition probabilities between CSTs across the workday (Fig. 3). The drivers starting with CST 1 in the morning primarily remained in CST 1 after the first unloading (88%) (Fig. 3a). Of the drivers who started with CST 2 in the morning, 10% remained in this CST after first unloading, while 90% switched to CST 1 (Fig. 3a). Forty-three percent of the drivers starting with CST 3 retained CST 3 also after first unloading (Fig. 3a). The remaining 57% of drivers switched from CST 3 to 1 (Fig. 3a). From first to second unloading, 85% of samples in CST 1 remained in CST 1 (Fig. 3b). All samples representing CST 2 and 3 after first unloading switched to CST 1 after second unloading, while only a few switched the other way (4% from CST 1 to CST 2; 12% from CST 1 to CST 3) (Fig. 3b). The few drivers’ samples that grouped into CST 2 or 3 after second unloading still belonged to the same CST the next morning (Fig. 3c). The majority of those samples with CST 1 after second unloading returned to either CST 2 (45%) or to CST 3 (27%) the next morning (Fig. 3c).

### LA-MRSA CC398 lineage dynamics in relation to nasal microbiota dynamics.

**FIG 4:**
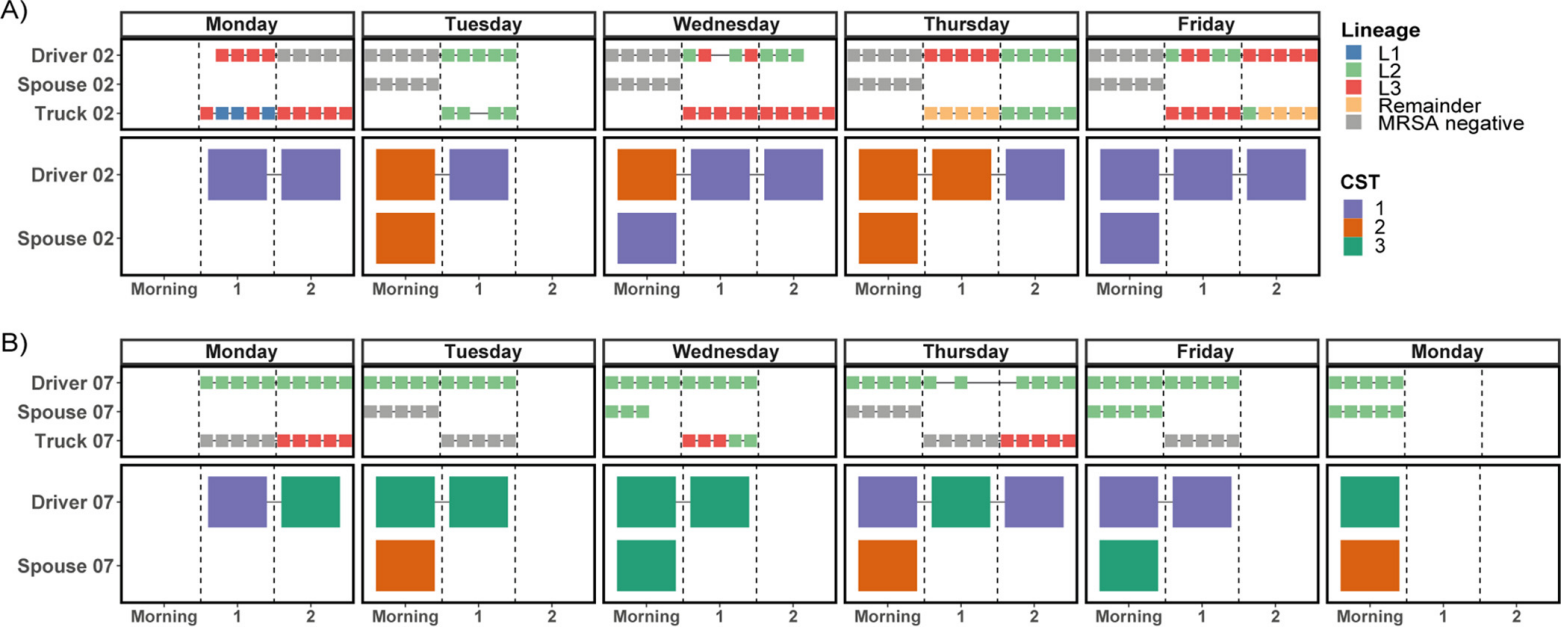
Temporal trajectories of LA-MRSA CC398 lineage associations and nasal CSTs. Top, lineages of MRSA isolates based on WGS; bottom, CSTs of the entire nasal microbiota based on 16S rRNA gene sequencing. (A) Example of an intermittent MRSA carrier (driver 02) and the corresponding spouse (spouse 02) and truck (truck 02). (B) Example of a persistent MRSA carrier (driver 07) and the corresponding spouse (spouse 07) and truck (truck 07). From each time point, 2 to 5 isolates were genome sequenced for those two households. Abbreviations: 1, time point after first unloading; 2, time point after second unloading; CST, community state type; WGS, whole-genome sequencing.

We performed WGS of LA-MRSA CC398 culture-positive isolates from the two persistent and six intermittent MRSA carriers (drivers), their spouses, and trucks in order to determine lineage affiliations and dynamics. Multiple isolates (2 to 5) per time point were sequenced from one persistent carrier (driver 07) and one intermittent carrier (driver 02) as well as from their spouses and trucks (Fig. 4). Sequences of one isolate per time point were obtained from the remaining six drivers. In total, 232 isolates from 103 samples were subjected to WGS (*n* = 69 from drivers, *n* = 4 from spouses, and *n* = 30 from trucks).

A phylogenetic tree depicts lineage affiliations (L1 to L3 and remainder) of the 232 isolates in relation to 288 previously published LA-MRSA CC398 isolates from Denmark and 88 CC398 isolates from an international reference collection (see Fig. S4 in the supplemental material) (3, 13). Subject-wise timelines of lineage affiliations extracted from the phylogenetic tree are shown in Fig. 4 and Fig. S5 in the supplemental material.

Because of the lineage ambiguity between multiple isolates within a time point observed for intermittent carrier driver 02, we prioritized describing the trajectories of driver 02 and driver 07 for whom whole-genome sequences for multiple isolates per time point are available. The intermittent carrier driver 02 was always MRSA negative in the morning (samples available for four mornings) and so was his spouse (spouse 02, Fig. 4a). On three of those mornings (75%), the nasal microbiota of driver 02 clustered into CST 2, which was the CST with the lowest staphylococcal density (Fig. 2). Spouse 02 grouped into either CST 1 or 2, matching the CST affiliation of driver 02 at three out of four mornings (75%) (Fig. 4a). After the first and second unloading, driver 02 was MRSA positive at eight out of nine available sampling time points (89%), with a lineage affiliation to either L2 or L3 (50% each). Wednesday and Friday after first unloading, isolates from the same time points showed ambiguity concerning lineage affiliation, with isolates from the same time point grouping into either L2 or L3. Eighty-nine percent of samples after first or second unloading were affiliated with CST 1 concerning their nasal microbial composition (Fig. 4a). Samples from truck 02 were collected only after the first and second unloading. Lineage affiliation of truck 02 isolates was ambiguous at two time points. At four out of nine available time points (44%), all or some truck isolates reflected the lineage affiliation of the corresponding driver’s isolates. However, at two time points, truck 02 clustered to none of the lineages (remainder), and at one time point, three isolates belonged to L1, which was not found among any of the MRSA isolates from driver 02 (Fig. 4a).

The persistent carrier driver 07 was MRSA positive at all sampled time points (Fig. 4b). All isolates were affiliated with lineage L2, and thus, there was no lineage ambiguity between isolates within any time point (Fig. 4b). Sixty percent of morning samples and 57% of samples after first and second unloading belonged to CST 3, which exhibited the highest density of the genus Staphylococcus (Fig. 4b and Fig. 2). Spouse 07 was MRSA positive at three out of five mornings (60%) and always affiliated with L2 (Fig. 4b). The spouse’s nasal microbiota clustered into CST 2 or 3 and matched the driver’s CST only once. Truck 07 was MRSA positive at three out of seven time points after unloading (43%), with isolates belonging predominantly to L3 (Fig. 4b).

Lineage trajectories of the remaining drivers with one isolate per time point (which does not account for potential lineage ambiguity) are shown in Fig. S5. Like driver 02, three out of the five remaining intermittent MRSA carriers were always negative in the mornings, while one was always positive (Fig. S5a). Their bacterial CST affiliation was not concordant, e.g., two intermittent carriers always grouped into CST 3 in the morning, while another always grouped into CST 2 (Fig. S5b). After the first and second unloading, the five intermittent carriers were MRSA positive (predominantly L2 and L3) at on average 82% of time points and mostly associated with CST 1 (88.4%) (Fig. S5). Overall, only one out of five spouses (spouse 09) of the intermittent carriers was MRSA positive at a single time point (Tuesday morning) (Fig. S5a). The lineage affiliation (L3) matched that of driver 09 at the same time point. Of note, the two drivers (07 and 09) with intermittently MRSA-positive spouses had the highest average MRSA CFU/ml (>10^5^) (Table 1).

## DISCUSSION

The present study describes LA-MRSA CC398 carriage and nasal microbiota dynamics in pig truck drivers, a hitherto understudied group within the pig production industry. Our findings highlight the importance of focusing on this occupation group which could represent potential LA-MRSA transmission routes to the community and hospitals due to their daily exposure to pigs from multiple farms.

We found a 40% prevalence of LA-MRSA CC398 in pig truck drivers, with 11% exhibiting CFU values above 1000 MRSA/swab. These numbers are far lower than that of pig farm workers for whom a prevalence of 63% (with 38% persistent MRSA carriers) in the Netherlands and 87% in Denmark has previously been shown (10, 14).

In our study, nasal bacterial alpha diversity in nonexposed individuals was higher than that in drivers (in the morning and after first unloading) and their spouses. At the end of the workday, alpha diversity of drivers increased to levels comparable to that of nonexposed subjects. However, comparing beta diversity of nonexposed subjects and drivers at the end of the workday showed highly different bacterial compositions, characterized by distinct taxa identified by LEfSe and clustering into CSTs. Although the nasal microbiota composition of nonexposed subjects differed from that of both spouses and drivers at all daily time points, it differed most from driver samples after pig contact. This finding indicates that the nasal microbiota is enriched with pig-associated taxa directly after pig exposure and does not fully return overnight to a microbiota comparable to that of nonexposed individuals. Therefore, pig truck drivers seem to harbor a persistently altered nasal microbiota, and it would be interesting to investigate whether a certain period of time without pig exposure could reverse this effect, similar to what has been observed for short-term visitors of pig farms (10). Moreover, the total bacterial load in the drivers’ nasal cavities was extremely high compared to that of nonexposed subjects. Part of this difference might be due to swabbing of drivers in both nostrils, while only one nostril was sampled in nonexposed individuals. Still, these high bacterial loads suggest that the nasal microbiota in truck drivers is permanently complemented by high loads of pig farm-associated bacteria in addition to common human nasal colonizers.

All but one of the nonexposed individuals in our study had a nasal microbiota characterized by *Corynebacterium* and *Dolosigranulum* (LEfSe/CST 2), which is in line with known healthy nasal colonization (15). Moreover, the most abundant staphylococcal species in CST 2 was most likely S. epidermidis, a common commensal of the human nose (16). In the morning, few drivers and the majority of spouses harbored the same CST as nonexposed subjects, although with higher relative abundances of Staphylococcus. However, most drivers started the workday with a clear S. aureus-dominated (CST 3) or *Psychrobacter*-dominated nasal microbiota (CST 1), supporting our finding that their microbiota does not fully reconstitute overnight after recurrent pig contact. Of note, species affiliation of the most abundant staphylococcal ASVs by BLAST may be ambiguous since the 16S rRNA (V3-V4) gene region of an S. aureus ASV prevalent in the human nose and skin has previously been described as being identical to that of Staphylococcus schweitzeri and Staphylococcus argenteus (0 single nucleotide polymorphism [SNP] differences) (17). Likewise, an S. epidermidis ASV had 0 SNP differences in the V3-V4 region compared with Staphylococcus caprae (17). S. aureus and S. epidermidis are however known to be more abundant in the human nose than *S. schweitzeri*, *S. argenteus*, and *S. caprae* (12). A reduced proportion of *Corynebacteriaceae* in pig-exposed compared with nonexposed individuals as revealed by LEfSe is in agreement with previous findings (18). Staphylococcal growth can be inhibited by certain *Corynebacterium* spp. so that a lack thereof might favor the staphylococcal dominance we observed (15). In the present study, during the course of the workday, most drivers switched to the *Psychrobacter*-dominated CST 1 after pig contact, which is in line with an overrepresentation of this genus found by LEfSe in drivers after first unloading. The high abundance of *Psychrobacter* was somewhat unexpected since this genus is usually associated with marine environments and only to a limited degree with terrestrial environments or homeothermic hosts (19). However, members of the *Psychrobacter* genus have previously been found in pig slurry and the pig gut, nose, and skin (20–22). *Psychrobacter* spp. were absent from our blank extraction controls and occurred primarily in drivers’ samples after contact with pigs; therefore, we can rule out a systematic laboratory contamination. Instead, we assume that *Psychrobacter* (family *Moraxellaceae*) originates from exposure of the drivers to pigs. Congruently, *Moraxellaceae* have previously been described as a dominating taxonomic family in pig nares (18). Other taxa dominating the drivers’ noses after pig contact, including *Clostridium* and *Bacillus*, have also been described as abundant in either the pig nose or pig farm dust, supporting the hypothesis of pig truck drivers acquiring a pig environment-like nasal microbiota during the workday (11, 22, 23).

We investigated associations between the nasal microbiota and LA-MRSA CC398 carrier status of the pig truck drivers. Most drivers were intermittent carriers who were mostly MRSA negative in the mornings, unlike pig farm workers who have been found to mostly retain MRSA overnight (10). When MRSA negative, the intermittent MRSA carrier for which several isolates per time point were available (driver 02) often concurrently clustered into CST 2, i.e., resembled the microbiota of nonexposed subjects. In light of the known association of S. aureus colonization and nasal microbial composition, this result could indicate that a microbiota similar to that of nonexposed subjects inhibits MRSA colonization or vice versa that nasal microbiota homeostasis benefits from the absence of S. aureus (12). However, overall, when drivers were MRSA negative, they were more often associated with CST 1 than CST 2. Despite intermittent carriers acquiring MRSA during the workday, the S. aureus-dominated CST 3 was less prevalent at the end of the workday. This finding is in line with a previous study on pig farm workers primarily clustering into CSTs with a low abundance of Staphylococcus in favor of highly abundant other pig-associated bacteria at the end of the workday (10). However, in the present study, absolute staphylococcal abundance in samples dominated by pig-associated bacteria (CST 1) still exceeded that of nonexposed individuals.

The persistent MRSA carriers often clustered into the S. aureus-dominated CST 3, especially in the morning. This result is in line with the previously described association of higher staphylococcal loads and persistent carrier status (12). The drivers carried primarily LA-MRSA lineages L2 and L3. The persistent carrier for whom multiple isolates per time point were available (driver 07) was solely colonized by L2, while intermittent carrier driver 02 could harbor L2 and L3 simultaneously. This finding indicates that driver 07 was persistently colonized by L2, which outcompeted other contaminating lineages. In contrast, the lineage ambiguity in driver 02 suggests that in intermittent carriers, several lineages transiently contaminate the nose, rather than colonizing it. Lineage L2 has previously been described as particularly prone to being spread through human carriers, and L3 was found to be the most rapidly spreading and most abundant lineage in Danish pig farms (3). This highlights the potential risk of LA-MRSA transmission between farms through pig truck drivers.

As an approximation for assessing LA-MRSA transmission to the community, we screened the drivers’ spouses. We found only a limited spread of LA-MRSA CC398 from drivers to spouses, and it was observed only from drivers with high bacterial loads of LA-MRSA CC398. Spouses of intermittent carriers were not colonized with MRSA, except for one spouse at a single time point. Only one spouse of a persistent carrier participated in the study and was MRSA positive at 60% of screened time points with the same lineage (L2) as the driver. This result might indicate that persistent carriage poses a greater risk of MRSA transmission to household and possibly community members than intermittent carriage, although this hypothesis is based on few observations. However, previous research found that the prevalence of MRSA in household members of pig farmers did not depend on whether the farmer was a persistent or intermittent carrier (31% versus 30%) (24). Importantly, household members of pig farmers are also directly exposed to the pig environment themselves in contrast to spouses of pig truck drivers. Since our observation is based on very few cases, further research is required.

Interestingly, some spouses did reflect their partner’s nasal microbiota CST at the same or following time points. While the majority of spouses clustered into CST 2, thereby resembling the microbiota of nonexposed individuals, some switched to the pig microbiota-like CST 1 or the S. aureus-dominated CST 3 matching their partners. This finding points to a risk of pig truck drivers introducing opportunistic livestock-associated pathogens other than S. aureus into their spouses’ microbiota which could lead to a possible route of transmission into the community or hospitals. However, the similarity of the microbiota of some spouses to that of the drivers also shows that spouses are not an optimal approximation of nonexposed individuals. Sharing a household with a pig truck driver and/or living in a rural area seem to impact the spouses’ nasal microbiota significantly. Therefore, it is limited to what extent MRSA transmission dynamics from drivers to spouses can be extrapolated to transmission dynamics to the community.

We found lower LA-MRSA prevalence in trucks than in drivers, likely due to extensive cleaning and disinfection of the trucks after each unloading. Cleaning between farm visits is an important biosecurity intervention, as previous research has shown MRSA transmission through trucks to the transported pigs (8, 9). Moreover, the low prevalence could be attributable to sample collection in five different places inside the truck at each time point which might be exposed to pigs to different degrees. Generally, the MRSA lineages found in the trucks matched those found in drivers to some extent. Trucks exhibited higher lineage diversity and more frequent switches between lineages, including all lineages (L1 to L3) as well as isolates not belonging to these lineages (remainder). Therefore, the trucks are expected to reflect the diverse lineage distribution on pig farms. One might therefore speculate that drivers picked up lineages L2 and L3 easier than L1 through contact with the truck and pigs.

Our study was limited by a relatively small number of study participants, as well as the lack of samples directly from pigs or pig farms. This limitation is, however, partly redeemed by sampling the pig trucks. With regard to the WGS analysis, the lineage ambiguity in those samples from which multiple isolates were sequenced indicates that sampling time points with only a single sequenced isolate is not reliable enough for determining lineage affiliations and concluding upon carriage dynamics. Another limitation was that we do not know whether nonexposed individuals live in the city or in the countryside and whether they may have contact with pigs. However, they were sampled in the urban capital region of Denmark, while the places of employment of the pig truck drivers are situated in rural areas of Denmark. Among individuals without direct contact with livestock, a previous study showed a higher incidence of LA-MRSA CC398 bloodstream infections for people living in rural areas (5). Therefore, the spouses in our study who share rural residency with pig truck drivers are not optimally representing community-dwelling individuals without livestock exposure.

In summary, 40% of pig truck drivers were intermittent or persistent carriers of LA-MRSA CC398. Especially, persistent carriers could be of risk to transmit MRSA to household members and further into the community and hospitals. However, we observed very limited transmission of LA-MRSA CC398 from drivers to spouses. We described associations between MRSA carrier status, the nasal microbiota composition, and bacterial and staphylococcal load in pig truck drivers. Our findings highlight that the nasal microbial composition could influence MRSA dynamics and vice versa. This influence might also be important in relation to the possible uptake of opportunistic pathogens other than LA-MRSA CC398 from the pig environment into the nasal microbiota.

## MATERIALS AND METHODS

### Study participants.

Initially, nasal swabs were collected from 47 pig truck drivers working at commercial slaughterhouses in Denmark and investigated for the presence of MRSA on 1 April 2019. The study was performed in accordance with principles of the Declaration of Helsinki and was approved by the Danish National Committee on Health Research Ethics (protocol H-15013814).

Nine of the MRSA-positive truck drivers volunteered to participate in the present study, conducted from 8 April to 15 April 2019. All driver participants were male with an average age of 45 years (range, 29 to 55 years) and have had close contact with pigs (private or professional) for an average of 20.8 years (range, 2 months to 50 years). Furthermore, spouses of six drivers were included in the study (average age, 44 years; range, 32 to 53); none of them had any contact with pigs. None of the participants were exposed to antibiotics during the study period.

### Sample collection.

Bacterial swab samples of drivers and spouses were collected from the anterior part of the nose using eSwabs (Copan, Brescia, Italy) by rotating the same swab five times in both nostrils. Nine pig truck drivers were sampled three times daily (in the morning before work, after the first unloading of pigs, and after the second unloading of pigs) for 6 days (Monday to Friday and the following Monday). On the first Monday, no morning samples were collected, while on the last Monday, only a morning sample was collected. Thus, there were 15 sampling time points in total. This way, we obtained 113 nasal samples from drivers (Table 1). The spouses of six drivers were sampled each morning (*n* = 25 samples). In the morning, drivers and spouses swabbed themselves at home according to our instructions and transported the sample in the fridge of their truck. Nasal swabs at the slaughterhouse were collected by an investigator. In addition, samples were taken from the pig trucks after each unloading of pigs (*n* = 73 samples). From each truck, five cotton swabs were used to collect debris from the inside the trucks and transferred to a tube with 10 ml of tryptic soy broth (Sigma-Aldrich) supplemented with 6.5% NaCl. After each transport, the truck was washed with water and detergent and thereafter sprayed with oxidative disinfectants (Fig. 1a). The trucks were kept closed until the next group of pigs were loaded in order to achieve maximal time of effect for the disinfectant.

All samples collected during a working day were refrigerated while stored and transported to the laboratory. Cultivation was performed the day after sampling. Samples collected on a Friday were cultivated the following Monday. Subsequently, samples were stored at −80°C.

### Cultivation of MRSA.

From each driver, spouse, and truck sample, MRSA was quantified by making serial dilutions of the swab fluid (1 ml) with 0.9% NaCl and 0.1% Triton X-100 (Sigma-Aldrich). Next, a 100-μl aliquot was spread onto one Brilliance MRSA 2 agar plate (Oxoid) and incubated at 35°C for 22 to 24 hours. Furthermore, all samples that were negative by direct cultivation were investigated for MRSA by enrichment in tryptic soy broth (Sigma-Aldrich) supplemented with 6.5% NaCl at 35°C for 16 to 24 hours, followed by spread of 10 μl onto Brilliance MRSA 2 agar plates, and incubation at 35°C for 22 to 24 hours. MRSA was identified as denim blue colonies. If possible, five colonies from each sample were selected for molecular verification.

All MRSA subcultures were verified by a PCR assay detecting *mecA* (giving resistance to methicillin), *lukF*-PV, *scn*, CC398-specific *sau1*-*hsdS1*, and *spa* followed by *spa* typing (25). LA-MRSA was identified by the presence of the S. aureus-specific *spa*, the *mecA* indicative for production of the Penicillin Binding Protein (PBP) 2A causing the MRSA phenotype and presence of the CC398-specific CC398*-hsdS1*, and absence of the human immune escape cluster amplicons.

### Whole-genome sequencing and data processing.

LA-MRSA CC398 isolates (*n* = 232) from culture-positive samples (*n* = 193) were whole-genome sequenced as described before (3). The resulting sequencing data were combined and further processed together with a previously sequenced international reference collection of 88 S. aureus CC398 isolates and a Danish reference collection of 288 isolates (3, 13). The sequence type 398 (ST398) reference genome (strain S0385; GenBank accession no. AM990992) was used for sequence read mapping and SNP calling by NASP version 1.0.2a1 (26). Subsequently, Gubbins (version 2.3.4) was used to remove regions of putative recombination from the alignment (27). A maximum-likelihood phylogenetic tree was then inferred using IQ-TREE (version 2.0.3) with default settings (28). The tree was rooted according to the international reference collection (13). The final phylogenetic tree was visualized in R with package ggtree (version 2.2.4; Fig. S4) (29). Samples of the present study were clustered into three LA-MRSA CC398 lineages (L1, L2, and L3) which have been previously defined based on the Danish reference collection (3).

### DNA isolation and 16S rRNA gene sequencing.

Nasal samples from drivers and their spouses were subjected to characterization of the nasal microbiota by 16S rRNA gene sequencing. Before DNA extraction, a 200-μl sample volume (out of 1 ml eSwab liquid amies) was used for enzymatic prelysis by incubation for 30 min at 37°C with enzyme solution containing 2.5 U lysostaphine [SAE0091], 25 U mutanolysin [sae0092], and 3 mg lysozyme ([L4919]) (Sigma-Aldrich, St. Louis, MO, USA), followed by a 30-min incubation at 56°C with 20 μl protein kinase K (RPROTKSOL-RO; Sigma-Aldrich). DNA from samples and blank controls was then extracted on a MagNA Pure 96 robot using a DNA and viral nucleic acid (NA) small volume kit (Roche, Mannheim, Germany). A mock community was used as positive controls (ZymoBiomics microbial community standard, D6300; Zymo Research).

The V3-V4 region of the 16S rRNA gene was amplified in a PCR using the following barcoded primers: 341F, 5′-CCTACGGGNGGCWGCAG-3′; and 805R, 5′-GACTACHVGGGTATCTAATCC-3′ (30). The primers were preceded by heterogeneity spacers. Library preparation was done using a Nextera XT DNA library preparation kit (Illumina Inc., San Diego, CA, USA). A MiSeq instrument (Illumina Inc.) was used for sequencing with a 600-cycle V3 kit.

### 16S rRNA gene sequence preprocessing.

Demultiplexing of raw reads was carried out by using the *bcl2fastq* conversion software (Illumina Inc., San Diego, CA, USA). Subsequently, *cutadapt* (version 2.3) was used to trim off heterogeneity spacers and primers at an error rate of 8% (corresponding to 1 mismatch per primer) (31). We then subjected the trimmed reads to *dada2* (version 1.12.1) to infer high-resolution amplicon sequence variants (ASVs) (32). Dada2 was run with default settings, except for truncation lengths which were adjusted to 270 bp for forward reads and 210 bp for reverse reads. Consensus chimera removal from the entire data set was performed. Taxonomic assignment was performed using the functions assignTaxonomy and addSpecies (R package DADA2) with the Silva reference database and species-level training set (v. 132) formatted for DADA2 (49).

During computational contaminant assessment with method “either” (prevalence threshold, 0.1; frequency threshold, 0.01) from the decontam R package, 43 ASVs were identified as potential contaminants and excluded (33). In addition, 514 ASVs were excluded which were either not classified up to at least order-level or belonged to *Archaea*, *Cyanobacteria*, *Planctomycetes*, *Chloroflexi*, *Deinococcus-Thermus*, *Rhizobiales*, *Rhodobacterales*, *Rhodospirillales*, *Oceanospirillales*, or *Rhodovibrionales*.

The final data set contained 230 samples with 5,889 unique ASVs and a median read count of 59,867 (range, 7,229 to 153,023).

### 16S rRNA gene qPCR.

For 16S rRNA gene quantitative PCR (qPCR), the universal 16S rRNA gene primers 16SF (5′-CCTACGGGNGGCWGCAG-3′) and 16SR (5′-GACTACHVGGGTATCTAATCC-3′) (Illumina Inc., San Diego, CA, USA) were used with a slightly modified version of the 16S-TQM-528R TaqMan probe (5′-6-carboxyfluorescein [FAM]-CGTATTACCGCGGCTGCTGGCAC-black hole quencher [BHQ]-1-3′) (34). The total reaction volume of 20 μl comprised 10 μl of 2× PerfeCTa qPCR ToughMix low ROX (Quanta BioSciences Inc., Gaithersburg, MD, USA), 1 mM dithiothreitol (DTT), and 0.4 U recombinant dsDNase (ArcticZymes, Tromsø, Norway) for potential elimination of PCR reagent bacterial DNA contaminants. The nuclease was activated by incubating the mixture at 37°C for 20 min and subsequently inactivated by incubating at 60°C for 20 min before 2 μl of the template was added. After dsDNase inactivation, the reagents were kept on a cold block in order to minimize the generation of primer-dimers due to partial activation of the *Taq* polymerase by the 60°C incubation step. The PCR was run on the ABI 7500 real-time PCR system (Applied Biosystems, Foster City, CA, USA) with 50 cycles of 95°C melting for 15 s, 55°C annealing for 30 s, and 72°C extension for 30 s. Data collection was at 72°C. A standard curve was generated from 10-fold dilutions of Legionella pneumophila purified genomic DNA ranging from 1.2 × 10^8^ to 1,000 genome equivalents per well, and the quantity of bacterial DNA in the samples was determined from the regression line. Samples exceeding the highest qPCR standard curve threshold of 1.2 × 10^8^ were diluted 10-fold. Molecular-grade water was used as a negative control in all PCRs.

### Inclusion of data from nonexposed adults.

Data from a previously published cohort of 92 healthy community-dwelling adults (referred to as nonexposed) recruited at the Danish national blood donor clinic at Hvidovre Hospital, Copenhagen, Denmark, in May 2019 and January 2020 were included in the study (35). Samples were collected at the anterior nares once (by rotating the swab three times in one nostril). These samples were kept in a box with dry ice for up to 6 hours before being stored at −80°C. DNA extraction, 16S rRNA gene (V3V4) sequencing, and qPCR were performed using the same protocols as those used for the other samples in this study. 16S rRNA gene sequences are available at the European Nucleotide Archive (ENA) (project number PRJEB42898). Three of the nonexposed individuals were excluded due to antibiotic treatment within 3 months prior to sampling, resulting in a final set of 89 nonexposed subjects (median age, 41 years; range, 18 to 66 years; 43 males [48%], 46 females [52%]).

### Statistical analysis to characterize the nasal microbiota.

Statistical analyses were performed in R (version 4.0.1) (36). ASV count table, taxonomy, and metadata were integrated in phyloseq (version 1.32.0.) (37). Visualizations were done with ggplot2 (version 3.3.1.), ggalluvial (version 0.11.3.), diagram (version 1.6.4.), and cowplot (version 1.0.0.) (38–41). We estimated nasal bacterial alpha diversity from raw counts by the Shannon index. For each time point (morning, after first/second unloading), we assessed in linear mixed models (LMMs) whether driver bacterial alpha diversity varied by weekday (with adjustment for repeated measurements per subject by inclusion as a random effect) (Fig. S1). Driver bacterial alpha diversity between time points within a day (morning, after first/second unloading) was then compared in an LMM (with adjustment for repeated measurements per subject and weekday by including those parameters as random effects). LMMs were computed using the package lmerTest (version 3.1.2.) with subsequent contrast analyses including false discovery rate (FDR) correction for multiple testing using the modelbased (version 0.1.2.) package (42, 43). Shannon diversity between drivers, spouses, and nonexposed subjects was compared using pairwise Wilcoxon signed-rank tests with FDR correction.

The count data were Hellinger transformed and subjected to PCoA. Differences in bacterial community structure between groups were compared by analysis of similarities (ANOSIM) from package vegan (version 2.5.6.) based on Bray-Curtis dissimilarities (44). In ANOSIM, dissimilarities between samples are ranked (thereby making it a nonparametric method), and it is assessed whether the mean ranked dissimilarities are greater between groups than within groups. The difference between groups is indicated by the ANOSIM statistic R. An R value close to 1 indicates that the bacterial composition of two groups is different, while an R value close to 0 indicates very similar compositions in the two groups. In general, the higher R, the more different the composition between the compared groups.

Differentially abundant taxa between time points during the day (drivers) and between drivers, spouses, and nonexposed individuals were identified by LEfSe on count data normalized to the sum of 1e + 06 with an LDA cutoff of 4 (package microbiomeMarker) (45). LEfSe accounts for the hierarchical structure of bacterial phylogeny, thereby allowing identification of differentially abundant taxa on several taxonomic levels (here, kingdom to genus).

Samples from drivers, spouses, and nonexposed subjects were grouped into community state types (CSTs) by partitioning around medoid (PAM) clustering based on the Jensen-Shannon distance with the R package cluster (version 2.1.0.) (46). The optimal number of clusters was predetermined based on consensus of the silhouette width, gap statistic, and elbow method within the factoextra package (version 1.0.7.) (47). For drivers, CST switches across the workday were visualized in a Sankey plot (complete cases only) and as Markov chains (Fig. 3). For the latter, transition probabilities between CSTs were calculated using the R package markovchain (version 0.8.5.) (48).

Total bacterial load per sample was calculated from the number of 16S rRNA gene copies (determined by qPCR) in 2 μl of DNA by upscaling to a 100-μl elution volume after DNA extraction and upscaling from a 200-μl input volume of liquid amies for DNA extraction to a 1-ml liquid amies volume in the swab collection tube. Absolute abundance of the genus Staphylococcus was estimated by multiplying sample-wise staphylococcal proportions with the number of total 16S rRNA gene copies per sample. This calculation did not account for potential copy number variations of the 16S rRNA gene between species. We assessed in LMMs whether the drivers’ overall bacterial and staphylococcal load varied by time point within a day (with adjustment for repeated measurements per subject and weekday by inclusion as random effects). Bacterial and staphylococcal load between drivers and spouses was compared using pairwise Wilcoxon signed-rank tests with FDR correction. We did not apply statistical tests for comparison with nonexposed individuals since those participants were swabbed in one nostril, while drivers were swabbed in both nostrils, which might to a certain extent lead to lower abundance in nonexposed individuals.

### Data availability.

The 16S rRNA gene sequences and genome sequences from drivers, spouses, and trucks are available through ENA at the European Bioinformatics Institute (EBI) under project number PRJEB43023. The data and R code used for statistical analyses are available from github (https://github.com/ssi-dk/Pig_truck_drivers_Ingham_et_al). Analysis output reports are available from figshare (https://figshare.com/projects/Dynamics_of_the_human_nasal_microbiota_and_Staphylococcus_aureus_CC398_carriage_in_pig_truck_drivers_across_one_workweek/98933).
