# Supplementary material for: Dynamics of the Human Nasal Microbiota and Staphylococcus aureus CC398 Carriage in Pig Truck Drivers across One Workweek
Source: Appl Environ Microbiol. 2021 Aug 26;87(18):e01225-21. doi: 10.1128/AEM.01225-21 (PMC8388827; doi:10.1128/AEM.01225-21)
Supplement: Supplemental file 1 — Figures S1 to S5, Table S1. Download AEM.01225-21-s0001.pdf, PDF file, 1.9 MB [file aem.01225-21-s0001.pdf]

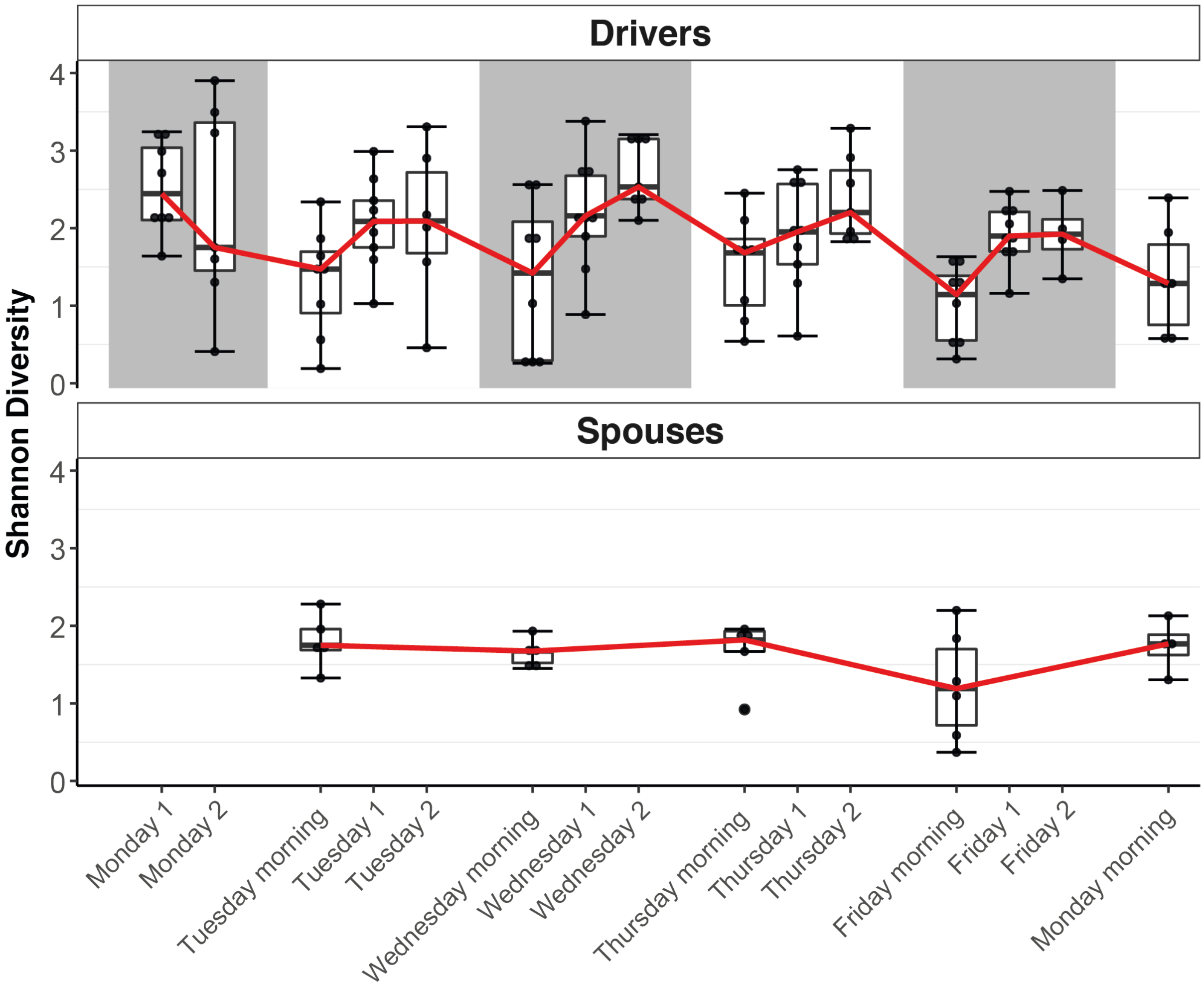

Supplemental Figure 1: Nasal bacterial alpha diversity across one workweek in pig truck drivers and spouses. Drivers' daily fluctuations in alpha diversity do not differ by weekday. Samples from spouses were only collected in the mornings. Abbreviations: '1': Timepoint after first unloading; '2': Timepoint after second unloading.

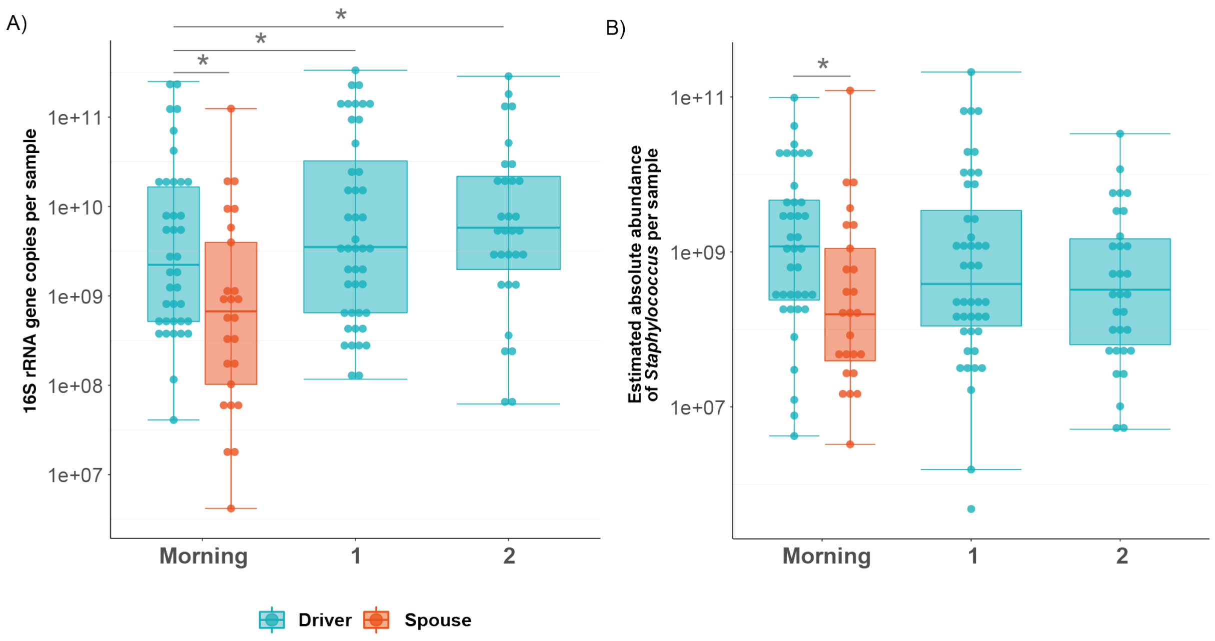

Supplemental Figure 2: Absolute abundance determined by qPCR. A) Total bacterial load per sample, calculated by upscaling from the qPCR input volume of 2 µl DNA to 100 µl elution volume after DNA extraction, and upscaling from 200 µl input volume of liquid amies for DNA extraction to 1 ml liquid amies volume in the swab collection tube. B) Staphylococcal load per sample, calculated from sample-wise relative abundances of the genus *Staphylococcus*. Abbreviations: '1': Timepoint after first unloading; '2': Timepoint after second unloading. . Asterisks indicate the following significance levels: * p<0.05; ** p<0.01; and *** p<0.001.

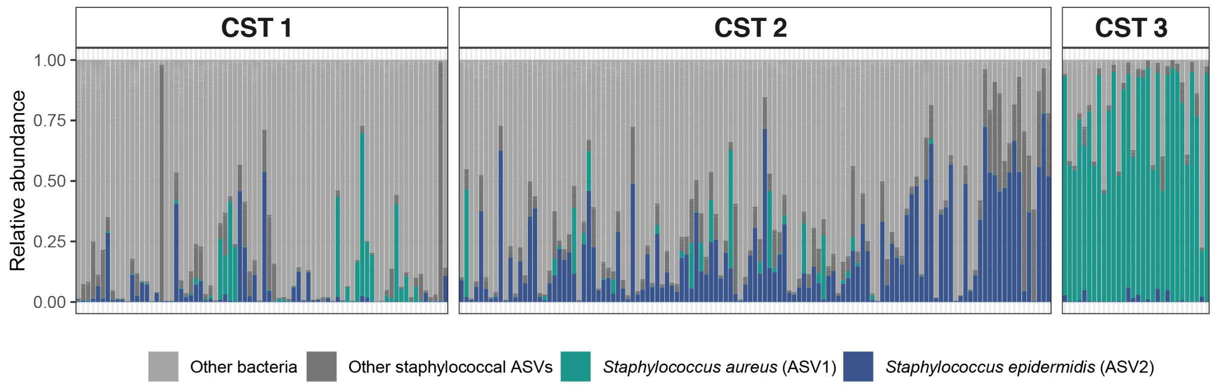

Supplemental Figure 3: Relative abundance of major staphylococcal ASVs per CST. The two most abundant ASV of the genus *Staphylococcus* were identified to be most likely *Staphylococcus aureus* (ASV1) and *Staphylococcus epidermidis* (ASV2) by a BLAST search. Staphylococcal dominance in CST3 was mainly attributable to *S. aureus* ASV1. Median relative abundance of *S. aureus* ASV1 was 0.2%, 0.1% and 80.2% in CST 1, 2, and 3, respectively. Median relative abundance of *S. epidermidis* ASV2 was highest in CST 2 with 0.7%, 12.9% and 0.2% in CST 1, 2, and 3, respectively. Abbreviations: CST: community state type.; ASV: amplicon sequence variant; BLAST: basic local alignment search tool.

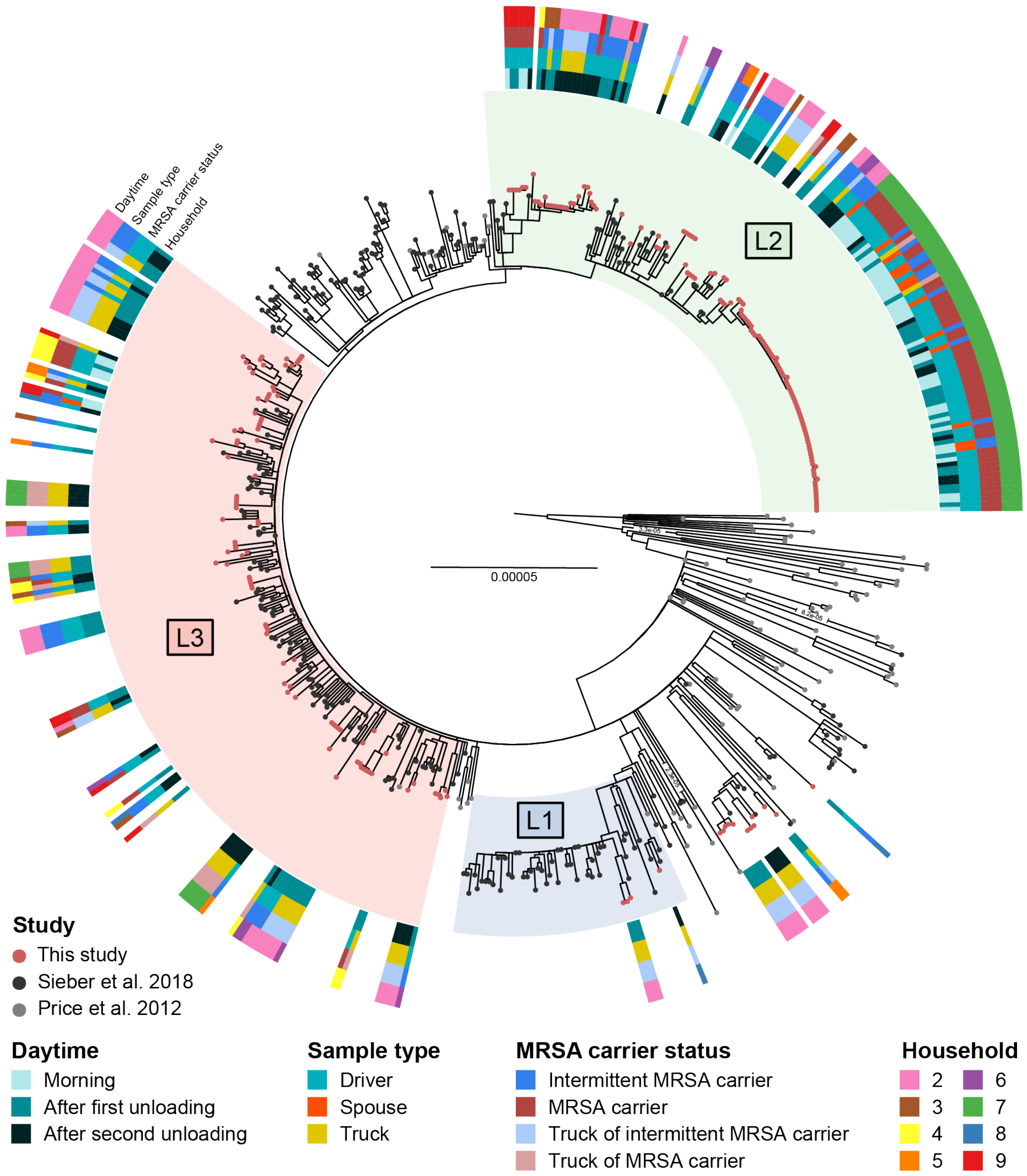

Supplemental Figure 4: Phylogenetic tree based on maximum likelihood. The tree includes 232 LA-MRSA CC398 isolates from drivers, spouses and trucks of this study in addition to 288 isolates from a Danish and 88 isolates from an international reference collection (1, 2). Multiple isolates per time point were sequenced for samples from households 2 and 7, while one isolate per time point was sequenced for samples from the remaining households. The scale bar represents the number of nucleotide substitutions per variable site. The tree was rooted according to the international reference collection (1). Abbreviations: LA-MRSA: livestock-associated methicillin-resistant *Staphylococcus aureus*; CC: clonal complex; L1: lineage 1; L2: lineage 2; L3: lineage 3.

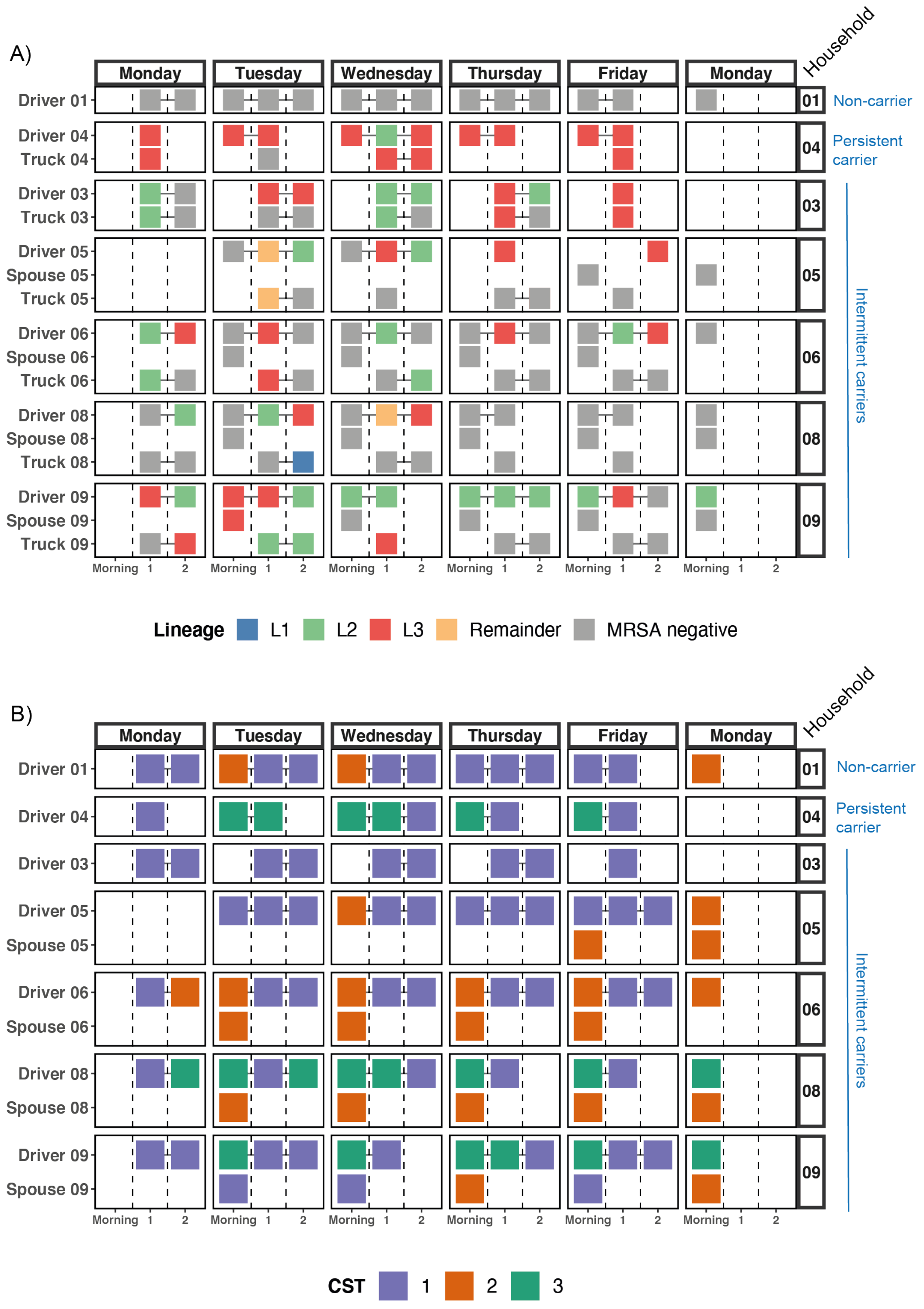

Supplemental Figure 5: Temporal trajectories of nasal CSTs and LA-MRSA CC398 lineage associations for subjects with one isolate per timepoint. A) Trajectories of LA-MRSA CC398 lineage associations based on WGS. B) Nasal microbial CST trajectories of drivers and spouses based on 16S rRNA sequencing. Abbreviations: '1': Timepoint after first unloading; '2': Timepoint after second unloading; CST: community state type; L1: lineage 1; L2: lineage 2; L3: lineage 3.

Supplemental Table S1: List of differentially abundant taxa identified by LEfSe. Taxa with a minimum LDA score of 4 are listed. Abbreviations: p: phylum, c: class, o: order, f: family, g: genus, LDA: Linear discriminant analysis, LEfSe: LDA effect size.

**Taxon**	**Enrich group**	**LDA score (log10)**	**adjusted p-value**
g__*Carnobacterium*	Spouse	4.5	2.35e-06
g__*Staphylococcus*	Driver - Morning	5.4	3.37e-09
f__*Staphylococcaceae*	Driver - Morning	5.4	8.81e-09
o__*Enterobacteriales*	Driver - Morning	4.7	9.96e-08
f__*Enterobacteriaceae*	Driver - Morning	4.7	9.96e-08
g__*Psychrobacter*	Driver – After 1. unloading	5.3	1.25e-39
g__*Rahnella*	Driver – After 1. unloading	4.4	8.34e-17
f__*Clostridiaceae 1*	Driver – After 1. unloading	4.0	4.04e-24
g__*Clostridium sensu stricto 1*	Driver – After 1. unloading	4.0	1.99e-25
f__*Enterococcaceae*	Driver – After 2. unloading	4.0	6.55e-08
c__*Actinobacteria*	Non-exposed	5.2	3.19e-28
p__*Actinobacteria*	Non-exposed	5.2	3.36e-28
o__*Corynebacteriales*	Non-exposed	5.2	9.08e-27
f__*Corynebacteriaceae*	Non-exposed	5.2	9.20e-27
g__*Corynebacterium 1*	Non-exposed	5.1	2.05e-25
c__*Clostridia*	Non-exposed	4.6	1.34e-15
o__*Clostridiales*	Non-exposed	4.6	1.34e-15
f__*Clostridiales* Family XI	Non-exposed	4.5	1.44e-22
g__*Anaerococcus*	Non-exposed	4.2	4.64e-21
g__*Lawsonella*	Non-exposed	4.2	3.61e-22
o__*Propionibacteriales*	Non-exposed	4.1	4.94e-36
f__*Propionibacteriaceae*	Non-exposed	4.1	4.80e-36
g__*Cutibacterium*	Non-exposed	4.1	4.78e-36

Supplemental references

1. Price LB, Stegger M, Hasman H, Aziz M, Larsen J, Andersen PS, Pearson T, Waters AE, Foster JT, Schupp J, Gillece J, Driebe E, Liu CM, Springer B, Zdovc I, Battisti A, Franco A, Zmudzki J, Schwarz S, Butaye P, Jouy E, Pomba C, Porrero MC, Ruimy R, Smith TC, Robinson DA, Weese JS, Arriola CS, Yu F, Laurent F, Keim P, Skov R, Aarestrup FM. 2012. Staphylococcus aureus CC398: Host adaptation and emergence of methicillin resistance in livestock. MBio 3:1–6.
2. Sieber RN, Skov RL, Nielsen J, Schulz J, Price LB, Larsen AR, Stegger M. 2018. Drivers and Dynamics of Methicillin-Resistant Livestock- Associated Staphylococcus aureus CC398 in Pigs and Humans in Denmark 9:1–12.
